# Renal replacement therapy in acute kidney injury: controversy and consensus

**DOI:** 10.1186/s13054-015-0850-8

**Published:** 2015-04-06

**Authors:** Claudio Ronco, Zaccaria Ricci, Daniel De Backer, John A Kellum, Fabio S Taccone, Michael Joannidis, Peter Pickkers, Vincenzo Cantaluppi, Franco Turani, Patrick Saudan, Rinaldo Bellomo, Olivier Joannes-Boyau, Massimo Antonelli, Didier Payen, John R Prowle, Jean-Louis Vincent

**Affiliations:** Department Nephrology Dialysis & Transplantation, International Renal Research Institute (IRRIV), San Bortolo Hospital, Viale Rodolfi, 36100 Vicenza, Italy; Department of Cardiology and Cardiac Surgery, Pediatric Cardiac Intensive Care Unit, Bambino Gesù Children’s Hospital, IRCCS, Piazza S. Onofrio 4, 00165, Rome, Italy; Department of Intensive Care, Erasme Hospital, Université libre de Bruxelles, Route de Lennik 808, 1070 Brussels, Belgium; Center for Critical Care Nephrology, Department of Critical Care Medicine, University of Pittsburgh, 3550 Terrace Street, Pittsburgh, PA 15261 USA; Division of Emergency and Intensive Care Medicine, Department of Internal Medicine, Medical University of Innsbruck, Anichstraße 35, 6020 Innsbruck, Austria; Department of Intensive Care Medicine, Radboud University Medical Centre, PO Box 9101, 6500 HB Nijmegen, The Netherlands; Nephrology, Dialysis and Kidney Transplantation Unit, University of Torino, Azienda Ospedaliera Universitaria ‘Città della Salute e della Scienza di Torino Presidio Molinette’, Corso Bramante 88, 10126 Turin, Italy; Department of Intensive Care, Aurelia Hospital and European Hospital, Via Portuense 694, 00416 Rome, Italy; Service of Nephrology, Department of Internal Medicine Specialties, University Hospital of Geneva, 4 rue Gabrielle Perret-Gentil, CH 1211 Geneva, Switzerland; Department of Intensive Care, Austin Hospital, 145 Studley Road, Heidelberg, Melbourne, VIC 3084 Australia; Centre Hospitalier Universitaire (CHU) de Bordeaux, Service d’Anesthésie-Réanimation 2, Avenue de Magellan, F-33600, Pessac, France; Università Cattolica del Sacro Cuore - Policlinico Universitario A. Gemelli, Largo Agostino Gemelli 8, 00168 Rome, Italy; Department of Anesthesiology and Critical Care, Lariboisière Hospital, Assistance Publique-Hôpitaux de Paris, University of Paris, 7 Denis Diderot, 75475, Paris, Cedex 10 France; Adult Critical Care Unit, The Royal London Hospital, Barts Health, Whitechapel Road, London, E1 1BB UK

## Abstract

Renal replacement therapies (RRTs) represent a cornerstone in the management of severe acute kidney injury. This area of intensive care and nephrology has undergone significant improvement and evolution in recent years. Continuous RRTs have been a major focus of new technological and treatment strategies. RRT is being used increasingly in the intensive care unit, not only for renal indications but also for other organ-supportive strategies. Several aspects related to RRT are now well established, but others remain controversial. In this review, we review the available RRT modalities, covering technical and clinical aspects. We discuss several controversial issues, provide some practical recommendations, and where possible suggest a research agenda for the future.

## Introduction

Acute kidney injury (AKI) is common in critically ill patients and is associated with high mortality and morbidity [1]. In patients with severe AKI, renal replacement therapy (RRT) represents a cornerstone of treatment. Although much progress has been made in this area, many questions remain unanswered [2].

The aim of this review is to present a multidimensional update of RRT in AKI with a focus on mechanisms and modalities, anticoagulation, and extended indications. The topics of plasmapheresis and RRT during extracorporeal membrane oxygenation will not be covered in this review.

## Principles of modern renal replacement therapy

Current RRT machines present multiple integrated components, including four- to five-roller pumps, three to four integrated scales, four to five pressure pods, reliable air detection, integrated pumps for heparin or citrate/calcium infusion, ultrafiltration, and various safety alarms. Considerable effort has gone into providing a user-friendly interface, limited error ranges, and possibility for data download/integration to electronic health records. New machines are often more a platform for multiple techniques than a simple dialysis monitor.

### Extracorporeal circuit

Extracorporeal therapies are now exclusively delivered through double-lumen venous catheters. With few exceptions, blood flow is ensured by a peristaltic roller pump. Circuit pressures are constantly monitored in every part of the circuit (pre-pump or access pressure, pre-filter, effluent). Furthermore, pressure drop and transmembrane pressures are calculated by the machine in order to monitor the filter clotting process. The most important aspect of filter patency is the process of concentration polarization, which is the accumulation of particles (mostly of protein nature) in the inner part of the hollow fiber that leads to a progressive decrease in membrane permeability and performance [3,4]. Possible solutions to reduce filter clotting and deterioration in membrane permeability are maximization of the blood flow rate (Qb) and optimization of the ultrafiltration-to-blood flow ratio - filtration fraction (FF) percentage - for post-dilution. Today, Qb values of greater than 200 mL/minute are easily achievable, and FF percentages of less than 20% are advised. Other strategies, such as changes in the structure of the fiber (inner diameter) and in filter geometry (length and number of fibers), are manufacturer-dependent.

### Vascular access

Vascular access is a particularly important determinant of the quality of RRT [5]. A large trial found that the jugular and femoral sites were equivalent in terms of infectious complications; however, jugular vein insertion may be preferable in obese patients [6]. Insertion in the left jugular vein has been associated with greater rates of catheter dysfunction compared with the right jugular vein or femoral veins [7]. Deeper insertion of jugular catheters with positioning in the right atrium has an advantage in terms of filter life and may be associated with better performance [8]. The subclavian veins should be avoided when possible as there is a risk of thrombosis which, in the event of non-recovery, can jeopardize arteriovenous fistula placement for chronic dialysis. The catheter diameter ideally should be about one third of the vein diameter to minimize vessel thrombosis. Ultrasound-guided positioning of catheters is recommended [2].

### Pre- or post-dilution

Hemofiltration (HF) is a process that parallels what occurs in the human glomerulus [9]. The tubular function is mimicked by fluid reinfusion to compensate, in total or in part, for the amount of filtered fluid. Reinfusion guarantees restoration of hematocrit to previous values and ensures solute dilution in the blood. Reinfusion can be performed before (pre-dilution) or after (post-dilution) the filter. Pre-dilution HF is less efficient because the filtrate contains fewer solutes since the blood will have been diluted before entering the filter. Nevertheless, the lower hemoconcentration inside the filter reduces the risk of clotting. Post-dilution is the best way to estimate creatinine/urea clearance in HF because clearance equals effluent rate. In post-dilution HF, the phenomenon of concentration polarization may induce membrane fouling and filter clotting. If pre-dilution HF is selected, protein layer deposition is reduced, but the efficiency of the treatment is reduced and the dose is not linearly proportional to the effluent rate [9]. Clearance is calculated by the effluent rate corrected for the dilution factor. No clinical study has definitively addressed when pre- or post-dilution HF should be used, so this decision is largely a matter of local experience and preference.

### Ultrafiltration

Fluid overload is associated with adverse outcomes in critical illness [10] and often represents a primary indication for RRT. Capillary leak and hypoalbuminemia predispose patients to the accumulation of interstitial edema and slow intravascular refilling. After initial resuscitation, it is crucial to avoid unnecessary fluid accumulation to limit later requirements for fluid removal. Patients should be actively assessed for the presence of fluid overload, and the rate and final target of fluid removal must be carefully considered and frequently reassessed, whichever method is used to achieve this. Setting the rate of removal requires consideration of the severity of complications of fluid overload, anticipated fluid intake, expected rate of vascular refilling, and cardiovascular tolerance to transient reduction in intravascular volume due to ultrafiltration. Techniques such as bioimpedance analysis and relative blood volume monitoring may assist in setting targets for total volume and rate of fluid removal, although these techniques have not been widely applied in the intensive care unit (ICU) [11,12]. Of note, although many tools can be used to predict the response to fluid administration (such as pulse pressure variations or passive leg-raising), there are no good indicators to predict tolerance to fluid removal. A fluid removal trial (reverse fluid challenge [13]) is therefore often the only option while assessing cardiovascular tolerance with the available hemodynamic tools.

## Timing, mode, and dose of renal replacement therapy

### When to start renal replacement therapy

The timing of initiation of RRT remains controversial. It is clear that derangements of, for example, potassium, acid–base balance, pronounced azotemia, and fluid overload (called the ‘conventional criteria’ [14] for initiating RRT) need correction. However, clinicians have difficulty estimating the likelihood of recovery from evolving AKI and this complicates the decision to start RRT. Renal biomarkers may be helpful in determining which patients will recover renal function prior to [15] or after [16] the initiation of RRT. Also, an isovolumic ‘furosemide stress test’ may help predict which patients will progress to more severe AKI [17].

The decision when to start RRT is not merely academic but may impact on outcomes. Although it has been suggested that early application of RRT in patients with severe sepsis, irrespective of the presence of renal failure, might be beneficial (for example, by modifying the plasma concentrations of inflammatory mediators [18]), early ‘classic dose’ continuous veno-venous hemofiltration (CVVH) did not limit further organ damage and even prolonged the need for organ support [19]. Nevertheless, although early initiation of RRT is not clearly associated with benefit, avoiding or delaying RRT is associated with higher mortality and increased hospital/ICU lengths of stay [20-22]. Based on these data, no clear guidance as to when to start RRT can currently be given. In addition, the terms ‘early’ and ‘late’ RRT are subjective and there is no reference definition.

Interestingly, fluid overload may be a major outcome determinant for critically ill patients with AKI at continuous renal replacement therapy (CRRT) start [23]; it is possible that starting ultrafiltration when a lower degree of fluid accumulation has been reached and targeting a negative fluid balance in the first treatment hours may improve outcome. However, the only available data so far come from retrospective studies or *post hoc* analyses [23], so that it is impossible to recommend *a priori* at what level of fluid gain RRT should be started or the net ultrafiltration rate that should be prescribed; these factors should be tailored according to individual patient requirements.

### When to stop renal replacement therapy

There is an even greater paucity of data on when to stop CRRT. RRT can be stopped when there is sufficient improvement in renal function, but how this can be evaluated while the patient is still receiving RRT remains unclear. Current practice suggests measuring urine output and serum creatinine levels while on a constant dose of CRRT and calculating the endogenous creatinine clearance by using both the urine and serum concentrations of creatinine. Decisions to delay or stop the next RRT session may be easier for intermittent treatments. Observational studies have shown that the most significant predictor of successful termination of CRRT is urine output [24]. A urine output of more than 400 mL/day is a reasonable cutoff value, resulting in correct classification in 79% of patients [25]. Not surprisingly, this predictive ability can be negatively influenced by the use of diuretics. However, the furosemide-induced response in patients without immediate recovery of renal function within 24 hours after cessation of CRRT can help predict eventual renal recovery [26]. The precise level of endogenous creatinine clearance needed to allow discontinuation of renal support has not been established but is assumed to be between 15 and 20 mL/minute. The need for RRT reinstitution is associated with increased mortality [25], likely reflecting a marker of disease severity rather than indicating that too early discontinuation is harmful. Administration of furosemide after termination of RRT increases urinary volume and sodium excretion but does not shorten the duration of renal failure [27]. To date, no study has used a marker other than creatinine as a measure of renal function. The fact that serum neutrophil gelatinase-associated lipocalin (NGAL) concentrations decreased when serum creatinine levels started to increase following cardiac surgery [28] and that the plasma clearance of NGAL is only 5 mL/minute during CRRT [29] suggests that biomarkers that reflect renal damage or function and that are not cleared by the CRRT may be useful to detect improvements in renal function while on CRRT. In the Acute Renal Failure Trial Network (ATN) study [30], creatinine clearance was assessed (using a 6-hour urine collection) when urine output exceeded 30 mL/hour or a decrease in creatinine level occurred while on CRRT. Renal support was discontinued when the measured creatinine clearance exceeded 20 mL/minute and was left to the discretion of providers when in the range of 12 to 20 mL/minute [30]. This approach currently represents the most accurate means of estimating when to end RRT.

### Continuous versus intermittent techniques

Intermittent hemodialysis (IHD) was originally conceived as a treatment for acute renal failure, and CRRT was introduced as an alternative when IHD was contraindicated. Subsequently, CRRT has become a routine therapy for AKI in many countries but may not be available in some resource-limited settings. Intermittent therapy has advantages in facilitating rehabilitation and other aspects of therapy for some patients. However, when applied correctly, both continuous and intermittent modalities can achieve a satisfactory degree of metabolic control in most patients, and randomized controlled trials (RCTs) and meta-analyses have not shown a difference between modalities in terms of patient survival rates. The reasons for this lack of evidence may still lie in the selection of patients for the study populations, with many of the most severely ill patients excluded. Importantly, a systematic analysis of RRT modality and its effect on renal recovery from dialysis dependence in critically ill patients found that CRRT was associated with a higher rate of renal recovery compared with IHD [31], suggesting that the use of CRRT may be more cost-effective than previously thought [32,33], especially as the real cost of conventional IHD is significantly higher than previously estimated [34]. However, these results were limited to observational studies, and no difference was found when the analysis was restricted to randomized trials. There is a broad consensus that, compared with standard IHD, CRRT may be the optimal treatment for hemodynamically unstable patients [2], and IHD may be a more suitable option when patients have left or are soon to leave the ICU.

### Hybrid therapies

Various ‘hybrid therapies’ [35,36] or ‘prolonged (daily) intermittent RRTs’ have been proposed as intermediate forms of therapy between continuous and intermittent. These techniques use various approaches, including sustained low-efficiency (daily) dialysis (SLE(D)D) and extended daily dialysis. In these techniques, conventional IHD equipment is adapted to provide longer session durations with lower flows and efficiency [35-37]. Several trials have indicated that the degree of hemodynamic stability with SLED and CRRT is quite similar [36-38]. One small trial found no difference between SLED and CVVH in terms of mortality (the primary endpoint), but SLED was associated with shorter lengths of stay and duration of mechanical ventilation (secondary endpoints) [39]. Possible further advantages of SLED may be more rapid mobilization of patients, which may result in shorter ICU stays and more rapid convalescence. Controversy exists regarding the definition of SLED as reported session times vary from as short as 6 hours every other day, thus resembling standard IHD, to more than 12 hours every day, thus approaching CRRT. An RCT investigating intensified SLED aimed at blood urea nitrogen (BUN) levels of less than 45 mg/dL did not show improved outcome compared with daily SLED achieving an average BUN of 60 to 75 mg/dL [40]. Another potential problem with SLED relates to difficulty in optimizing doses of antibiotics. There is an increased risk of antibiotic underdosage during the second half of the SLED session. This may be particularly relevant for patients infected with multiresistant strains. Despite availability of these approaches, clinical use so far has been limited to only a relatively small number of centers.

### Dose of renal replacement therapy

The dose of RRT is equivalent to the normalized dose of therapy delivered to the patient. In the past, RRT was largely under-dosed in critically ill patients. However, since the milestone trial by the group of Vicenza in 2000 [41], a dose of 35 mL/kg per hour has been widely used, with a trend to increased doses in patients with sepsis [42].

Two large multicenter RCTs, the Randomized Evaluation of Normal versus Augmented Level of Renal Replacement (RENAL) and ATN studies [30,43], showed that increased intensity of RRT was not associated with improved patient outcomes. As a consequence, the recommended ‘normal dose’ is now in the range of 20 to 30 mL/kg per hour for continuous therapies. This observation does not mean that individual patients may not benefit from higher doses when hypercatabolic states or sepsis is present.

**Figure 1:**
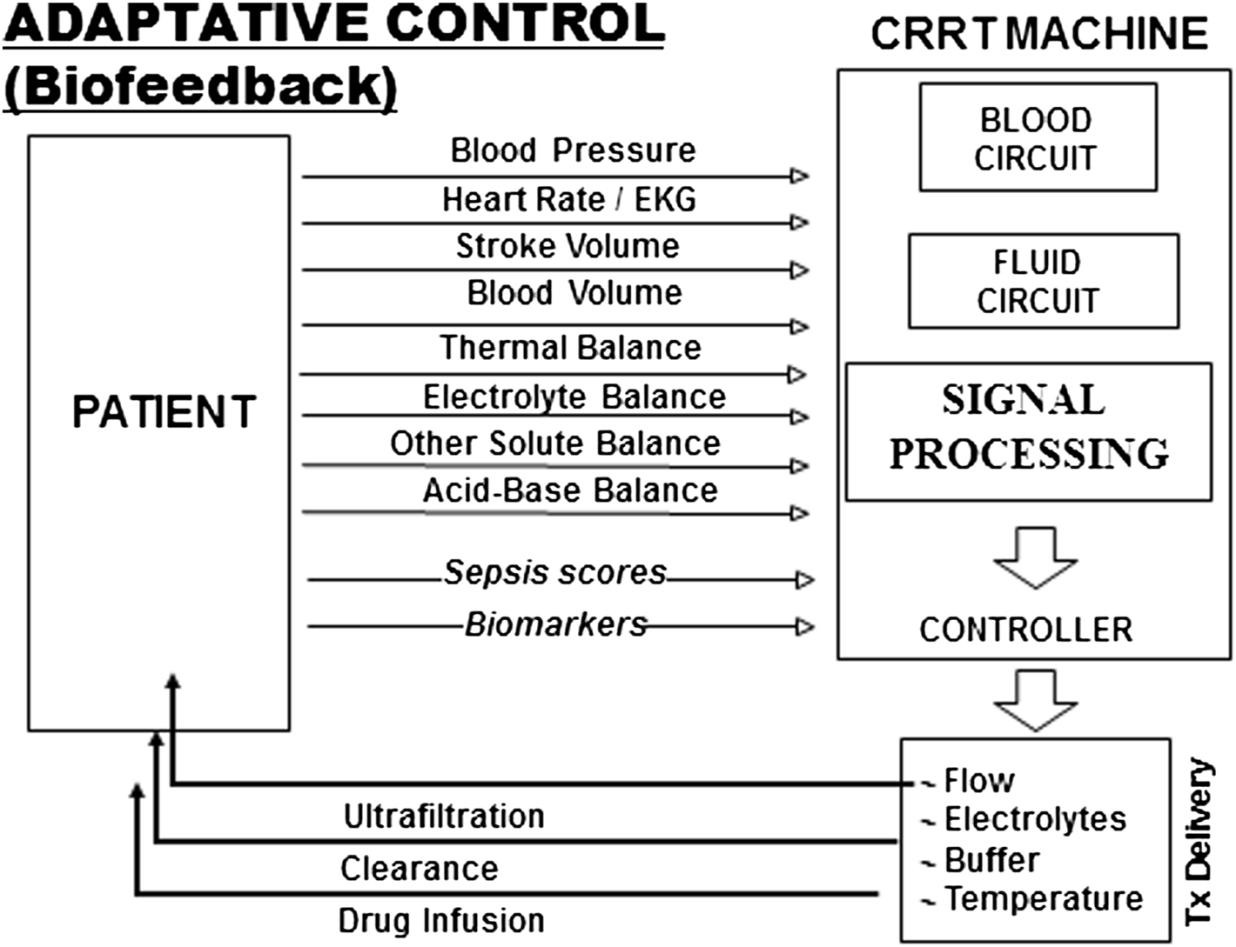
**The ‘ideal’ future renal replacement technology.** The ‘ideal’ future renal replacement technology will couple renal replacement therapy intensity (treatment delivery) with different bio-feedback systems to tailor dose and ultrafiltration rate to the complex needs of the individual critically ill patient. EKG, electrocardiogram; Tx, treatment.

In patients who had higher day 1 concentrations of plasma inflammatory mediators, intensive RRT was associated with reduced levels over the first week but no significant effect on renal recovery or mortality [44]. However, numerous studies have shown that persistently elevated concentrations of inflammatory markers are associated with RRT dependence and death [45,46]. These observations suggest that a personalized approach, mapping RRT intensity to biomarker levels, could be a more effective option. These effects, however, may be too small to translate into clinical benefit. Better technology is probably needed, possibly coupling RRT intensity with monitoring of different biomarkers over time (Figure 1).

## Potential complications

### Metabolic complications

The increased solute clearance achieved by CRRT may cause unwanted losses of amino acids, vitamins, catecholamines, and other compounds [47]. Severe hypophosphatemia (<1.0 mg/dL; <0.32 mmol/L) occurs in about half of ICU patients [48], and CRRT can contribute to this deficiency [49], especially when high-intensity treatment is prescribed. Hypophosphatemia is associated with respiratory and cardiac depression and immune dysfunction [50]. This condition should be prevented or adequately treated with supplementation, especially in CRRT patients. Phosphate-containing CRRT replacement fluids which protect against hypophosphatemia are now available [51]. Hypomagnesemia can also occur during CRRT as the replacement fluid also lacks magnesium.

### Anticoagulation

Sufficient anticoagulation, without excessive risk of bleeding, is generally required to maintain filter and circuit patency in RRT. Although the precise definition of adequate filter patency is a matter of debate, most available membranes will have significant protein concentration polarization and pore fouling after 20 to 24 hours. Point-of-care (POC) techniques of coagulation - for example, rotational thromboelastometry and thromboelastography - are increasingly being used for rapid specific testing of hemostatic function in critically ill patients and during anesthesia with important clinical benefits [52]. Nevertheless, very few studies have addressed the utility of POC techniques in critically ill patients during CRRT [53]. Because standard laboratory tests of blood coagulation may be of limited value to detect the changes during RRT, we anticipate that POC coagulation testing will be used more frequently during RRT, especially in septic patients, in whom early clotting of the cartridge may occur.

## Heparin, heparinoids, and thrombin inhibitors

Unfractionated heparin (UFH) is still the most widely used anticoagulant [54]. The major advantages of UFH are low costs, ease of administration, simple monitoring, and reversibility with protamine. The half-life of UFH is about 90 minutes, increasing to up to 3 hours in renal insufficiency because of accumulation of smaller fragments [55]. The activated partial thromboplastin time is still the best option for monitoring but may not be a reliable predictor of bleeding [56]; levels of greater than 45 to 50 seconds have been associated with an increased risk of bleeding [57]. At this low level of anticoagulation, the activated clotting time is relatively insensitive. A possible scheme for UFH consists of a bolus of 30 IU/kg followed by an initial rate of 5 to 10 IU/kg per hour in patients with normal coagulation. In addition to bleeding, other side effects of UFH include the development of heparin-induced thrombocytopenia (HIT) and effects on serum lipids; efficacy is also dependent on antithrombin levels, and substitution may be needed in patients with severe sepsis and heparin resistance [58].

Low-molecular-weight heparins (LMWHs) have several advantages over UFH, including a lower incidence of HIT, less affinity for antithrombin, less platelet (and polymorphonuclear cell) activation, less inactivation by platelet factor-4, greater and more consistent bioavailability, and no metabolic side effects [55]. However, LMWHs are eliminated by CRRT. Although some studies have used fixed doses of an LMWH, continuous intravenous administration of LMWH adjusted to achieve systemic anti-Factor Xa levels of 0.25 to 0.35 U/mL may be the safer option, resulting in improved filter survival as compared with UFH [59]. However, anti-Xa levels may not be a reliable predictor of bleeding [60]. Whenever HIT is diagnosed or suspected, all heparins should be discontinued and replaced by an alternative anticoagulant [61], such as danaparoid or a direct thrombin inhibitor (for example, bivalirudin or argatroban) (Table 1).

**Table 1:** **Alternative anticoagulation in heparin-induced thrombocytopenia** [115-119]

	**Danaparoid**	**Bivalirudin**	**Argatroban**
Dosing	3,500 IU bolus, followed by 100 units/hour or 140 IU/hour without bolus	0.03-0.2 mg/kg per hour	Bolus 100 *μ*g/kg followed by 0.1-0.5 μg/kg per minute
Monitoring	Anti-Xa activity 0.25-0.35 IU/mL (0.5-1.0 IU/mL^a^)	Target aPTT ratio 1.5 (−2.5^a^)	Target aPTT ratio 1.5 (−3.0^a^)
Main adverse events	Cross-reactivity with HIT-ab	No data	Anemia; accumulation in liver failure

^a^If systemic anticoagulation is required. aPTT, activated partial thromboplastin time; HIT-ab, heparin-incuded thrombocytopenia antibody.

## Regional citrate anticoagulation

The complications associated with systemic heparin administration stimulated interest in regional anticoagulation techniques and especially regional citrate anticoagulation (RCA) [62-64]. Sodium citrate is infused before the patient’s blood enters the CRRT circuit, and forms a complex with ionized calcium, removing this key component from the coagulation pathways. Extracorporeal calcium concentrations of less than 0.35 mmol/L are usually sufficient for regional anticoagulation, requiring citrate doses of approximately 4 to 6 mmol/L blood. Most of the calcium citrate complexes quickly pass the filter membrane and are lost in the effluent volume. The remaining calcium citrate enters the systemic circulation and is metabolized in the liver, muscle, and kidney, producing three molecules of bicarbonate for each molecule of citrate. Citrate, therefore, can have general metabolic consequences; for example, in the presence of severe liver failure, citrate accumulation may occur and is best detected by the total calcium-to-ionized calcium ratio - a ratio of more than 2.5 indicates citrate accumulation syndrome and treatment must be stopped. Additional calcium infusions compensate for extracorporeal losses in the circuit, maintaining the patient’s normal calcium levels [63].

Compared with systemic heparin, RCA has been associated with less bleeding, increased filter lifespan, and reduced transfusion rates and need for antithrombin III/platelet supplementation [65-67]. RCA has also been shown to have potential anti-inflammatory effects, including reduced degranulation of polymorphonuclear cells and increased formation of platelet-leukocyte complexes, oxidative stress, and interleukin-1-beta release [68-70]. The impact of RCA on outcome has not been clearly demonstrated [65,66,71],

Although KDIGO (Kidney Disease: Improving Global Outcomes) guidelines suggest that citrate be used for all patients without contraindications and heparin for other patients [72], some prefer to keep heparin as a first-choice treatment, especially for units using RRT less frequently, but to replace it with citrate in patients with a high risk of bleeding. As technological advances facilitate citrate use, we anticipate increased use.

### Antibiotic dosing during renal replacement therapy

Early and adequate antibiotic therapy is crucial in critically ill patients with signs and symptoms of infection. AKI alters antibiotic elimination, leading to drug accumulation [73], and the use of CRRT may further modify antibiotic pharmacokinetics [74,75]. However, current recommendations on antibiotic dosing during CRRT are based on studies that included a limited number of patients, that had varying inclusion/exclusion criteria, and that included patients who received different types of RRT [74,76,77]. Effluent flow rate was the most reliable predictor of antibiotic clearance in critically ill patients, and higher dosing regimens may be required in critically ill patients receiving RRT [78,79], in particular for poorly susceptible strains. Nevertheless, this strategy may also be associated with very high drug levels in the late phase of therapy, with the potential development of drug-related side effects [80]. When possible, therefore, antibiotic concentrations should be measured in patients undergoing CRRT and dosing schedules adapted to the individual patient.

## Renal replacement therapy as an adjunct in sepsis

Cytokines and other inflammatory mediators contribute to the pathogenesis of septic shock and sepsis-associated AKI [81]. However, although CRRT removes inflammatory cytokines and partially modulates plasma cytokines, outcomes do not seem to be affected regardless of the dose applied [19,82-84]. These findings may be due to the timing of implementation and patient selection. Moreover, whereas studies have found that inflammatory mediators vary widely in patients with sepsis [85], blood purification studies have generally not measured inflammation as part of the inclusion criteria.

Standard filtration or dialysis membranes - even when used with high-volume HF - have demonstrated only limited effectiveness in removing cytokines, the vast majority of which are water-soluble and of mid-range molecular weight (5 to 51 kDa) [84]. This is most likely attributable to the limited pore size of standard blood purification membranes. Recently, high cutoff (HCO) membranes with moderately larger pore sizes have been developed and found to have greater cytokine removal capacity compared with standard high-flux membranes in *ex vivo* experiments, animal experiments, and preliminary clinical studies [86,87]. Moreover, HCO membranes were associated with beneficial effects on immune cell function and increased survival in animal models of sepsis [88]. This technology may represent a valuable tool to attenuate the inflammatory response in patients with septic shock. Further studies of HCO RRT in the treatment of patients with septic shock appear warranted.

### Adsorption

It has been suggested that by separating the plasma so that cells do not come into contact with the sorbent, a system would need to be less hemocompatible but could be more effective. Coupled plasma filtration and adsorption (CPFA) separates plasma from blood by means of a plasma filter. The plasma is then passed through a synthetic resin cartridge for adsorption and returned to the blood. Unlike standard RRT, adsorption is very effective in removing large solutes. Once the blood has been reconstituted, a second blood filter can be used to remove excess fluid and small-molecular-weight toxins. CPFA and CRRT then can operate in series. A pilot clinical study using CPFA in sepsis demonstrated a marked resolution of immunoparalysis with function of circulating leukocytes restored to normal [89]. A recent study suggested that treating high volumes of plasma may be an important objective of CPFA, but this is difficult to achieve [90]. An RCT of high-dose CPFA in patients with septic shock is ongoing in several Italian centers (ClinicalTrials.gov identifier: NCT01639664).

### Polymyxin B hemoperfusion

Polymyxin B hemoperfusion has been shown to decrease macrophage and monocyte activity and to inactivate circulating pro-apoptotic factors potentially involved in the pathogenic mechanisms of sepsis-associated AKI [91,92]. A systematic review of available clinical studies suggested beneficial effects on arterial pressure, gas exchange, and even mortality [93]. In a recent meta-analysis [94], RRT, and especially hemoperfusion, was associated with improved survival in patients with sepsis. However, many of the studies were limited by suboptimal methodological quality. In EUPHAS (Early Use of Polymyxin B Hemoperfusion in Abdominal Sepsis), a prospective multicenter RCT in 64 patients with severe sepsis or septic shock who underwent emergency surgery for intra-abdominal infection, addition of polymyxin B hemoperfusion to standard therapy was associated with improved blood pressure, reduced organ failure, and possibly improved survival [95]. Final results from the ABDO-MIX study (ClinicalTrials.gov identifier: NCT01222663), a randomized multicenter study in patients with septic shock undergoing surgical treatment for peritonitis, have not yet been published, but there was no improvement in survival with polymyxin B hemoperfusion. The EUPHRATES trial (ClinicalTrials.gov identifier: NCT01046669) is ongoing in North America and hopefully will definitively evaluate the safety and efficacy of polymyxin B hemoperfusion in patients with septic shock.

## Renal replacement therapy in other conditions

### Cardiac failure and cardiorenal syndromes

Patients with cardiac failure have reduced cardiac output and arterial underfilling but also venous congestion. Both of these aspects may cause AKI by reducing renal perfusion pressure [96,97]. Mechanical ultrafiltration may be useful to resolve fluid overload by achieving better sodium removal per unit volume than diuretic therapy, thus resulting in better improvement of cardiovascular function. In the UNLOAD study [98], ultrafiltration compared with diuretics in patients with cardiac failure and impaired renal function resulted in lower rates of re-hospitalization, but the subsequent CARRESS (Effectiveness of Ultrafiltration in Treating People With Acute Decompensated Heart Failure and Cardiorenal Syndrome) study [99], in a similar population, was discontinued early with no evidence of benefit from ultrafiltration. However, in the CARRESS study, ultrafiltration was applied without clear evidence of diuretic resistance and fluid removal was set at a fixed rate for all patients and without the same level of hemodynamic support allowed in the pharmacological arm. Finally, in a case series of 63 chronic heart failure patients with diuretic-resistant fluid overload, treatment with slow continuous ultrafiltration was associated with an improvement in central pressures and cardiac index after 48 hours of treatment [100]. Outcomes were favorable in patients who did not require transition to RRT for solute clearance; however, only 8 of 37 who received RRT survived with recovered renal function. Thus, diuretic resistance and the need for RRT may be markers of the severity of chronic disease and of poor outcome where treatment options may be limited. Furthermore, HF, but not ultrafiltration, may allow sodium and water removal to be dissociated, with specific homeostatic benefits in acute cardiorenal syndromes.

### Respiratory failure

Respiratory failure is frequently associated with impaired renal function. Reduced cardiac output because of increased thoracic pressure results in activation of volume regulation characterized by sodium and water retention as well as reduced renal blood flow [101]. Fluid retention results in interstitial edema, which compromises pulmonary function by increasing extravascular lung water. The resulting increase in central venous pressure may play a significant role in reducing glomerular filtration rate [96]. Injurious mechanical ventilation is also characterized by a systemic release of cytokines, which is an additional risk factor for AKI [102]. AKI in turn results in decreased cytokine clearance and increased systemic release of inflammatory markers, resulting in increased alveolar fluid and a pulmonary inflammatory reaction, even in previously healthy lung [103]. Overall, this vicious circle tends to terminate in acute respiratory distress syndrome (ARDS) and AKI. Thus, applying protective ventilation using tidal volumes of around 6 mL/kg [104] may also be considered kidney-protective ventilation. In the presence of massive fluid overload and ARDS, CRRT may be considered to reduce extravascular lung water and allow less invasive ventilation. Combinations of RRT with extracorporeal CO_2_ removal techniques may help to re-establish acid–base homeostasis and reduce vasopressor demands as well as ventilation pressures. Though not as effective as extracorporeal membrane oxygenation, extracorporeal CO_2_ removal in series with CRRT may contribute to further reduce tidal volumes (ultraprotective ventilation) or even help to avoid intubation [105,106].

### Acute brain injury

AKI occurs in 8% to 23% of patients with acute brain injury and is an independent predictor of poor outcome in these patients [107,108]. AKI is associated with several complex pathological pathways, including sodium imbalance, alteration of nitric oxide synthase expression, and inhibition of gamma-aminobutyric acid neurotransmission, which can all result in brain injury; AKI is also associated with increased brain inflammation and increased blood–brain barrier (BBB) permeability [109]. Interestingly, the increased urea and solute levels in the blood also result in an intra-cerebral shift of these molecules as well as increased cerebral water content [110]; this process is more marked in patients with acute brain injury because of the disrupted BBB and altered aquaporin metabolism. Thus, when RRT is initiated in patients with acute brain injury, the decrease in serum osmolarity can create an osmotic gradient across the BBB, which will be compensated by a shift of water into the brain compartment, thus contributing to increase intracranial pressure (ICP) [111]. Another potentially adverse cerebral event associated with RRT in such patients is arterial hypotension, which could result in brain hypoperfusion. The use of bicarbonate buffers in RRT can also increase CO_2_ production and thus brain CO_2_ partial pressure, with cerebral vasodilation and risk of increased ICP.

CRRT should be the first option in these patients because it is associated with less increase in ICP compared with intermittent therapies [111]. CRRT is also associated with better maintenance of cerebral blood flow autoregulation after traumatic brain injury [112]. Because of the risk of cerebral hemorrhage with systemic heparin, RCA may be preferred [113]. Moreover, it has been suggested that citrate may provide some neuroprotection by attenuating hypoxic neuronal injury through its effects on astrocytes and oxidative phosphorylation [114].

## Conclusion: multiple organ support therapy

Critically ill patients often develop multiple organ dysfunction. New extracorporeal therapies are being designed to provide supportive treatment beyond the classic renal indications. In the near future, technical developments in extracorporeal devices will lead to the creation of multiple organ support therapies, so that comprehensive replacement or at least support can be provided to multiple organs simultaneously. New machines already include multiple platforms in which different circuits and filters can be used in combination to support renal, heart, liver, and lung function. Such machines ideally will be able to automatically detect both ‘traditional’ (urea) and ‘inflammatory’ (cytokines) solutes in plasma of critically ill patients in order to automatically (or semi-automatically) tailor the therapy toward the ‘perfect blood purification’ system (Figure 1). Along with technological advances, organizational issues at the institutional level, staff training, and operator knowledge of critical care nephrology need to be improved in order to optimize the safe and effective use of RRT.

## Abbreviations

AKI: Acute kidney injury
ARDS: Acute respiratory distress syndrome
ATN: Acute Renal Failure Trial Network
BBB: Blood–brain barrier
BUN: Blood urea nitrogen
CARRESS: Effectiveness of Ultrafiltration in Treating People With Acute Decompensated Heart Failure and Cardiorenal Syndrome
CPFA: Couple plasma filtration and adsorption
CRRT: Continuous renal replacement therapy
CVVH: Continuous veno-venous hemofiltration
FF: Filtration fraction
HCO: High cutoff
HF: Hemofiltration
HIT: Heparin-induced thrombocytopenia
ICP: Intracranial pressure
ICU: Intensive care unit
IHD: Intermittent hemodialysis
LMWH: Low-molecular-weight heparin
NGAL: Neutrophil gelatinase-associated lipocalin
POC: Point-of-care
Qb: Blood flow rate
RCA: Regional citrate anticoagulation
RCT: Randomized controlled trial
RRT: Renal replacement therapy
SLED: Sustained low-efficiency dialysis
UFH: Unfractionated heparin

## References

[1] Bellomo R, Ronco C, Ronco C, Bellomo R, Kellum JA (2009). Continuous renal replacement therapy: hemofiltration, hemodiafiltration or hemodialysis. Critcal Care Nephrology.

[2] Kidney Disease Outcomes Quality Initiative (2012). KDIGO Clinical Practice Guidelines for Acute Kidney Injury. Kidney Int Suppl.

[3] Ward D, Ronco C, Bellomo R, Kellum JA (2009). Principles of extracorporeal circulation. Critical Care Nephrology.

[4] Baldwin I, Bellomo R, Koch B (2004). Blood flow reductions during continuous renal replacement therapy and circuit life. Intensive Care Med.

[5] Vijayan A (2009). Vascular access for continuous renal replacement therapy. Semin Dial.

[6] Parienti JJ, Thirion M, Megarbane B, Souweine B, Ouchikhe A, Polito A, Forel JM, Marque S, Misset B, Airapetian N, Daurel C, Mira JP, Ramakers M, du Cheyron D, Le Coutour X, Daubin C, Charbonneau P (2008). Femoral vs jugular venous catheterization and risk of nosocomial events in adults requiring acute renal replacement therapy: a randomized controlled trial. JAMA.

[7] Parienti JJ, Megarbane B, Fischer MO, Lautrette A, Gazui N, Marin N, Hanouz JL, Ramakers M, Daubin C, Mira JP, Charbonneau P, du Cheyron D (2010). Catheter dysfunction and dialysis performance according to vascular access among 736 critically ill adults requiring renal replacement therapy: a randomized controlled study. Crit Care Med.

[8] Morgan D, Ho K, Murray C, Davies H, Louw J (2012). A randomized trial of catheters of different lengths to achieve right atrium versus superior vena cava placement for continuous renal replacement therapy. Am J Kidney Dis.

[9] Ricci Z, Ronco C (2005). Pre- versus post-dilution CVVH. Blood Purif.

[10] Payen D, de Pont AC, Sakr Y, Spies C, Reinhart K, Vincent JL (2008). A positive fluid balance is associated with a worse outcome in patients with acute renal failure. Crit Care.

[11] House AA, Haapio M, Lentini P, Bobek I, de Cal M, Cruz DN, Virzi GM, Carraro R, Gallo G, Piccinni P, Ronco C (2011). Volume assessment in mechanically ventilated critical care patients using bioimpedance vectorial analysis, brain natriuretic peptide, and central venous pressure. Int J Nephrol.

[12] Ronco C, Kaushik M, Valle R, Aspromonte N, Peacock WF (2012). Diagnosis and management of fluid overload in heart failure and cardio-renal syndrome: the ‘5B’ approach. Semin Nephrol.

[13] Hoste EA, Maitland K, Brudney CS, Mehta R, Vincent JL, Yates D, Kellum JA, Mythen MG, Shaw AD (2014). Four phases of intravenous fluid therapy: a conceptual model. Br J Anaesth.

[14] Bellomo R, Kellum JA, Ronco C (2012). Acute kidney injury. Lancet.

[15] Srisawat N, Murugan R, Lee M, Kong L, Carter M, Angus DC, Kellum JA (2011). Plasma neutrophil gelatinase-associated lipocalin predicts recovery from acute kidney injury following community-acquired pneumonia. Kidney Int.

[16] Srisawat N, Wen X, Lee M, Kong L, Elder M, Carter M, Unruh M, Finkel K, Vijayan A, Ramkumar M, Paganini E, Singbartl K, Palevsky PM, Kellum JA (2011). Urinary biomarkers and renal recovery in critically ill patients with renal support. Clin J Am Soc Nephrol.

[17] Chawla LS, Davison DL, Brasha-Mitchell E, Koyner JL, Arthur JM, Shaw AD, Tumlin JA, Trevino SA, Kimmel PL, Seneff MG (2013). Development and standardization of a furosemide stress test to predict the severity of acute kidney injury. Crit Care.

[18] Honore PM, Jamez J, Wauthier M, Lee PA, Dugernier T, Pirenne B, Hanique G, Matson JR (2000). Prospective evaluation of short-term, high-volume isovolemic hemofiltration on the hemodynamic course and outcome in patients with intractable circulatory failure resulting from septic shock. Crit Care Med.

[19] Payen D, Mateo J, Cavaillon JM, Fraisse F, Floriot C, Vicaut E (2009). Impact of continuous venovenous hemofiltration on organ failure during the early phase of severe sepsis: a randomized controlled trial. Crit Care Med.

[20] Karvellas CJ, Farhat MR, Sajjad I, Mogensen SS, Leung AA, Wald R, Bagshaw SM (2011). A comparison of early versus late initiation of renal replacement therapy in critically ill patients with acute kidney injury: a systematic review and meta-analysis. Crit Care.

[21] Leite TT, Macedo E, Pereira SM, Bandeira SR, Pontes PH, Garcia AS, Militao FR, Sobrinho IM, Assuncao LM, Liborio AB (2013). Timing of renal replacement therapy initiation by AKIN classification system. Crit Care.

[22] Clec’h C, Darmon M, Lautrette A, Chemouni F, Azoulay E, Schwebel C, Dumenil AS, Garrouste-Org, Goldgran-Toledano D, Cohen Y, Timsit JF (2012). Efficacy of renal replacement therapy in critically ill patients: a propensity analysis. Crit Care.

[23] Bellomo R, Cass A, Cole L, Finfer S, Gallagher M, Lee J, Lo S, McArthur C, McGuiness S, Norton R, Myburgh J, Scheinkestel C, Su S (2012). An observational study fluid balance and patient outcomes in the Randomized Evaluation of Normal vs Augmented Level of Replacement Therapy trial. Crit Care Med.

[24] Wu VC, Ko WJ, Chang HW, Chen YW, Lin YF, Shiao CC, Chen YM, Chen YS, Tsai PR, Hu FC, Wang JY, Lin YH, Wu KD (2008). Risk factors of early redialysis after weaning from postoperative acute renal replacement therapy. Intensive Care Med.

[25] Uchino S, Bellomo R, Morimatsu H, Morgera S, Schetz M, Tan I, Bouman C, Macedo E, Gibney N, Tolwani A, Straaten HO, Ronco C, Kellum JA (2009). Discontinuation of continuous renal replacement therapy: a post hoc analysis of a prospective multicenter observational study. Crit Care Med.

[26] van der Voort PH, Boerma E, Pickkers P (2014). The furosemide stress test to predict renal function after continuous renal replacement therapy. Crit Care.

[27] van der Voort PH, Boerma EC, Koopmans M, Zandberg M, de Ruiter J, Gerritsen RT, Egbers PH, Kingma WP, Kuiper MA (2009). Furosemide does not improve renal recovery after hemofiltration for acute renal failure in critically ill patients: a double blind randomized controlled trial. Crit Care Med.

[28] Mishra J, Dent C, Tarabishi R, Mitsnefes MM, Ma Q, Kelly C, Ruff SM, Zahedi K, Shao M, Bean J, Mori K, Barasch J, Devarajan P (2005). Neutrophil gelatinase-associated lipocalin (NGAL) as a biomarker for acute renal injury after cardiac surgery. Lancet.

[29] de Geus HR, Betjes MG, Bakker J (2010). Neutrophil gelatinase-associated lipocalin clearance during veno-venous continuous renal replacement therapy in critically ill patients. Intensive Care Med.

[30] Palevsky PM, Zhang JH, O’Connor TZ, Chertow GM, Crowley ST, Choudhury D, Finkel K, Kellum JA, Paganini E, Schein RM, Smith MW, Swanson KM, Thompson BT, Vijayan A, Watnick S, Star RA, Peduzzi P (2008). Intensity of renal support in critically ill patients with acute kidney injury. N Engl J Med.

[31] Schneider AG, Bellomo R, Bagshaw SM, Glassford NJ, Lo S, Jun M, Cass A, Gallagher M (2013). Choice of renal replacement therapy modality and dialysis dependence after acute kidney injury: a systematic review and meta-analysis. Intensive Care Med.

[32] Srisawat N, Lawsin L, Uchino S, Bellomo R, Kellum JA (2010). Cost of acute renal replacement therapy in the intensive care unit: results from The Beginning and Ending Supportive Therapy for the Kidney (BEST Kidney) study. Crit Care.

[33] Manns B, Doig CJ, Lee H, Dean S, Tonelli M, Johnson D, Donaldson C (2003). Cost of acute renal failure requiring dialysis in the intensive care unit: clinical and resource implications of renal recovery. Crit Care Med.

[34] Wald R, Shariff SZ, Adhikari NK, Bagshaw SM, Burns KE, Friedrich JO, Garg AX, Harel Z, Kitchlu A, Ray JG (2014). The association between renal replacement therapy modality and long-term outcomes among critically ill adults with acute kidney injury: a retrospective cohort study. Crit Care Med.

[35] Berbece AN, Richardson RM (2006). Sustained low-efficiency dialysis in the ICU: cost, anticoagulation, and solute removal. Kidney Int.

[36] Kumar VA, Craig M, Depner TA, Yeun JY (2000). Extended daily dialysis: a new approach to renal replacement for acute renal failure in the intensive care unit. Am J Kidney Dis.

[37] Wu VC, Wang CH, Wang WJ, Lin YF, Hu FC, Chen YW, Chen YS, Wu MS, Lin YH, Kuo CC, Huang TM, Chen YM, Tsai PR, Ko WJ, Wu KD (2010). Sustained low-efficiency dialysis versus continuous veno-venous hemofiltration for postsurgical acute renal failure. Am J Surg.

[38] Kielstein JT, Kretschmer U, Ernst T, Hafer C, Bahr MJ, Haller H, Fliser D (2004). Efficacy and cardiovascular tolerability of extended dialysis in critically ill patients: a randomized controlled study. Am J Kidney Dis.

[39] Schwenger V, Weigand MA, Hoffmann O, Dikow R, Kihm LP, Seckinger J, Miftari N, Schaier M, Hofer S, Haar C, Nawroth PP, Zeier M, Martin E, Morath C (2012). Sustained low efficiency dialysis using a single-pass batch system in acute kidney injury - a randomized interventional trial: the REnal Replacement Therapy Study in Intensive Care Unit PatiEnts. Crit Care.

[40] Faulhaber-Walter R, Hafer C, Jahr N, Vahlbruch J, Hoy L, Haller H, Fliser D, Kielstein JT (2009). The Hannover Dialysis Outcome study: comparison of standard versus intensified extended dialysis for treatment of patients with acute kidney injury in the intensive care unit. Nephrol Dial Transplant.

[41] Ronco C, Bellomo R, Homel P, Brendolan A, Dan M, Piccinni P, La Greca G (2000). Effects of different doses in continuous veno-venous haemofiltration on outcomes of acute renal failure: a prospective randomised trial. Lancet.

[42] Legrand M, Darmon M, Joannidis M, Payen D (2013). Management of renal replacement therapy in ICU patients: an international survey. Intensive Care Med.

[43] Bellomo R, Cass A, Cole L, Finfer S, Gallagher M, Lo S, McArthur C, McGuinness S, Myburgh J, Norton R, Scheinkestel C, Su S (2009). Intensity of continuous renal-replacement therapy in critically ill patients. N Engl J Med.

[44] Wen X, Murugan R, Kong L, Shan N, Lee MJ, Carter MJ, Elder M, Palevsky PM, Unruh M, Kellum JA (2011). Differential effects of renal replacement therapy on plasma inflammatory and apoptotic biomarkers in acute kidney injury [abstract]. J Am Soc Nephrol.

[45] Murugan R, Wen X, Shah N, Lee M, Kong L, Pike F, Keener C, Unruh M, Finkel K, Vijayan A, Palevsky PM, Paganini E, Carter M, Elder M, Kellum JA (2014). Plasma inflammatory and apoptosis markers are associated with dialysis dependence and death among critically ill patients receiving renal replacement therapy. Nephrol Dial Transplant.

[46] Payen D, Lukaszewicz AC, Legrand M, Gayat E, Faivre V, Megarbane B, Azoulay E, Fieux F, Charron D, Loiseau P, Busson M (2012). A multicentre study of acute kidney injury in severe sepsis and septic shock: association with inflammatory phenotype and HLA genotype. PLoS One.

[47] Btaiche IF, Mohammad RA, Alaniz C, Mueller BA (2008). Amino acid requirements in critically ill patients with acute kidney injury treated with continuous renal replacement therapy. Pharmacotherapy.

[48] Geerse DA, Bindels AJ, Kuiper MA, Roos AN, Spronk PE, Schultz MJ (2010). Treatment of hypophosphatemia in the intensive care unit: a review. Crit Care.

[49] Ricci Z, Salvatori G, Bonello M, Ratanarat R, Andrikos E, Dan M, Piccinni P, Ronco C (2005). A new machine for continuous renal replacement therapy: from development to clinical testing. Expert Rev Med Devices.

[50] Hiremath S, Slivar S, Magner P (2013). Phosphate balance with continuous renal replacement therapy: a simple solution. Am J Kidney Dis.

[51] Chua HR, Schneider AG, Baldwin I, Collins A, Ho L, Bellomo R (2013). Phoxilium vs Hemosol-B0 for continuous renal replacement therapy in acute kidney injury. J Crit Care.

[52] Weber CF, Gorlinger K, Meininger D, Herrmann E, Bingold T, Moritz A, Cohn LH, Zacharowski K (2012). Point-of-care testing: a prospective, randomized clinical trial of efficacy in coagulopathic cardiac surgery patients. Anesthesiology.

[53] Holt AW, Bierer P, Glover P, Plummer JL, Bersten AD (2002). Conventional coagulation and thromboelastograph parameters and longevity of continuous renal replacement circuits. Intensive Care Med.

[54] Hirsh J, Warkentin TE, Shaughnessy SG, Anand SS, Halperin JL, Raschke R, Granger C, Ohman EM, Dalen JE (2001). Heparin and low-molecular-weight heparin: mechanisms of action, pharmacokinetics, dosing, monitoring, efficacy, and safety. Chest.

[55] Joannidis M, Oudemans-van Straaten HM (2007). Clinical review: Patency of the circuit in continuous renal replacement therapy. Crit Care.

[56] Oudemans-van Straaten HM, Wester JP, de Pont AC, Schetz MR (2006). Anticoagulation strategies in continuous renal replacement therapy: can the choice be evidence based?. Intensive Care Med.

[57] van de Wetering J, Westendorp RG, van der Hoeven JG, Stolk B, Feuth JD, Chang PC (1996). Heparin use in continuous renal replacement procedures: the struggle between filter coagulation and patient hemorrhage. J Am Soc Nephrol.

[58] du Cheyron D, Bouchet B, Bruel C, Daubin C, Ramakers M, Charbonneau P (2006). Antithrombin supplementation for anticoagulation during continuous hemofiltration in critically ill patients with septic shock: a case–control study. Crit Care.

[59] Joannidis M, Kountchev J, Rauchenzauner M, Schusterschitz N, Ulmer H, Mayr A, Bellmann R (2007). Enoxaparin vs. unfractionated heparin for anticoagulation during continuous veno-venous hemofiltration: a randomized controlled crossover study. Intensive Care Med.

[60] Greaves M (2002). Limitations of the laboratory monitoring of heparin therapy. Scientific and Standardization Committee Communications: on behalf of the Control of Anticoagulation Subcommittee of the Scientific and Standardization Committee of the International Society of Thrombosis and Haemostasis. Thromb Haemost.

[61] Selleng K, Warkentin TE, Greinacher A (2007). Heparin-induced thrombocytopenia in intensive care patients. Crit Care Med.

[62] Oudemans-van Straaten HM, Kellum JA, Bellomo R (2011). Clinical review: anticoagulation for continuous renal replacement therapy - heparin or citrate?. Crit Care.

[63] Lanckohr C, Hahnenkamp K, Boschin M (2013). Continuous renal replacement therapy with regional citrate anticoagulation: do we really know the details?. Curr Opin Anaesthesiol.

[64] Tolwani A, Wille KM (2012). Advances in continuous renal replacement therapy: citrate anticoagulation update. Blood Purif.

[65] Hetzel GR, Schmitz M, Wissing H, Ries W, Schott G, Heering PJ, Isgro F, Kribben A, Himmele R, Grabensee B, Rump LC (2011). Regional citrate versus systemic heparin for anticoagulation in critically ill patients on continuous venovenous haemofiltration: a prospective randomized multicentre trial. Nephrol Dial Transplant.

[66] Oudemans-van Straaten HM, Bosman RJ, Koopmans M, van der Voort PH, Wester JP, van der Spoel JI, Dijksman LM, Zandstra DF (2009). Citrate anticoagulation for continuous venovenous hemofiltration. Crit Care Med.

[67] Morabito S, Pistolesi V, Tritapepe L, Zeppilli L, Polistena F, Strampelli E, Pierucci A (2012). Regional citrate anticoagulation in cardiac surgery patients at high risk of bleeding: a continuous veno-venous hemofiltration protocol with a low concentration citrate solution. Crit Care.

[68] Gritters M, Grooteman MP, Schoorl M, Schoorl M, Bartels PC, Scheffer PG, Teerlink T, Schalkwijk CG, Spreeuwenberg M, Nube MJ (2006). Citrate anticoagulation abolishes degranulation of polymorphonuclear cells and platelets and reduces oxidative stress during haemodialysis. Nephrol Dial Transplant.

[69] Gabutti L, Ferrari N, Mombelli G, Keller F, Marone C (2004). The favorable effect of regional citrate anticoagulation on interleukin-1beta release is dissociated from both coagulation and complement activation. J Nephrol.

[70] Bournazos S, Rennie J, Hart SP, Dransfield I (2008). Choice of anticoagulant critically affects measurement of circulating platelet-leukocyte complexes. Arterioscler Thromb Vasc Biol.

[71] Schilder L, Nurmohamed S, Bosch FH, Purmer IM, den Boer SS, Kleppe CG, Vervloet MG, Beishuizen A, Girbes A, Ter Wee PM, Groeneveld A (2014). Citrate anticoagulation versus systemic heparinisation in continuous venovenous hemofiltration in critically ill patients with acute kidney injury: a multi-center randomized clinical trial. Crit Care.

[72] Lameire N, Kellum JA (2013). Contrast-induced acute kidney injury and renal support for acute kidney injury: a KDIGO summary (Part 2). Crit Care.

[73] Blot S, Lipman J, Roberts DM, Roberts JA (2014). The influence of acute kidney injury on antimicrobial dosing in critically ill patients: are dose reductions always necessary?. Diagn Microbiol Infect Dis.

[74] Roberts DM, Roberts JA, Roberts MS, Liu X, Nair P, Cole L, Lipman J, Bellomo R (2012). Variability of antibiotic concentrations in critically ill patients receiving continuous renal replacement therapy: a multicentre pharmacokinetic study. Crit Care Med.

[75] Hites M, Dell’Anna AM, Scolletta S, Taccone FS (2014). The challenges of multiple organ dysfunction syndrome and extra-corporeal circuits for drug delivery in critically ill patients. Adv Drug Deliv Rev.

[76] Beumier M, Roberts JA, Kabtouri H, Hites M, Cotton F, Wolff F, Lipman J, Jacobs F, Vincent JL, Taccone FS (2013). A new regimen for continuous infusion of vancomycin during continuous renal replacement therapy. J Antimicrob Chemother.

[77] Taccone FS, De Backer D, Laterre PF, Spapen H, Dugernier T, Delattre I, Wallemacq P, Vincent JL, Jacobs F (2011). Pharmacokinetics of a loading dose of amikacin in septic patients undergoing continuous renal replacement therapy. Int J Antimicrob Agents.

[78] Jamal JA, Udy AA, Lipman J, Roberts JA (2014). The impact of variation in renal replacement therapy settings on piperacillin, meropenem, and vancomycin drug clearance in the critically ill: an analysis of published literature and dosing regimens. Crit Care Med.

[79] Seyler L, Cotton F, Taccone FS, De Backer D, Macours P, Vincent JL, Jacobs F (2011). Recommended beta-lactam regimens are inadequate in septic patients treated with continuous renal replacement therapy. Crit Care.

[80] Beumier M, Casu GS, Hites M, Seyler L, Cotton F, Vincent JL, Jacobs F, Taccone FS (2014). beta-lactam antibiotic concentrations during continuous renal replacement therapy. Crit Care.

[81] Zarjou A, Agarwal A (2011). Sepsis and acute kidney injury. J Am Soc Nephrol.

[82] Joannes-Boyau O, Honore PM, Perez P, Bagshaw SM, Grand H, Canivet JL, Dewitte A, Flamens C, Pujol W, Grandoulier AS, Fleureau C, Jacobs R, Broux C, Floch H, Branchard O, Franck S, Roze H, Collin V, Boer W, Calderon J, Gauche B, Spapen HD, Janvier G, Ouattara A (2013). High-volume versus standard-volume haemofiltration for septic shock patients with acute kidney injury (IVOIRE study): a multicentre randomized controlled trial. Intensive Care Med.

[83] Zhang P, Yang Y, Lv R, Zhang Y, Xie W, Chen J (2012). Effect of the intensity of continuous renal replacement therapy in patients with sepsis and acute kidney injury: a single-center randomized clinical trial. Nephrol Dial Transplant.

[84] Lehner GF, Wiedermann CJ, Joannidis M (2014). High-volume hemofiltration in critically ill patients: a systematic review and meta-analysis. Minerva Anestesiol.

[85] Kellum JA, Kong L, Fink MP, Weissfeld LA, Yealy DM, Pinsky MR, Fine J, Krichevsky A, Delude RL, Angus DC (2007). Understanding the inflammatory cytokine response in pneumonia and sepsis: results of the Genetic and Inflammatory Markers of Sepsis (GenIMS) Study. Arch Intern Med.

[86] Atan R, Crosbie D, Bellomo R (2012). Techniques of extracorporeal cytokine removal: a systematic review of the literature. Blood Purif.

[87] Atan R, Crosbie DC, Bellomo R (2013). Techniques of extracorporeal cytokine removal: a systematic review of human studies. Ren Fail.

[88] Haase M, Bellomo R, Morgera S, Baldwin I, Boyce N (2007). High cut-off point membranes in septic acute renal failure: a systematic review. Int J Artif Organs.

[89] Ronco C, Brendolan A, Lonnemann G, Bellomo R, Piccinni P, Digito A, Dan M, Irone M, La Greca G, Inguaggiato P, Maggiore U, De Nitti C, Wratten ML, Ricci Z, Tetta C (2002). A pilot study of coupled plasma filtration with adsorption in septic shock. Crit Care Med.

[90] Livigni S, Bertolini G, Rossi C, Ferrari F, Giardino M, Pozzato M, Remuzzi G (2014). Efficacy of coupled plasma filtration adsorption (CPFA) in patients with septic shock: a multicenter randomised controlled clinical trial. BMJ Open.

[91] Kushi H, Miki T, Sakagami Y, Sato J, Saito T, Tanjoh K (2009). Hemoperfusion with an immobilized polymyxin B fiber column decreases macrophage and monocyte activity. Ther Apher Dial.

[92] Cantaluppi V, Assenzio B, Pasero D, Romanazzi GM, Pacitti A, Lanfranco G, Puntorieri V, Martin EL, Mascia L, Monti G, Casella G, Segoloni GP, Camussi G, Ranieri VM (2008). Polymyxin-B hemoperfusion inactivates circulating proapoptotic factors. Intensive Care Med.

[93] Cruz DN, Perazella MA, Bellomo R, de Cal M, Polanco N, Corradi V, Lentini P, Nalesso F, Ueno T, Ranieri VM, Ronco C (2007). Effectiveness of polymyxin B-immobilized fiber column in sepsis: a systematic review. Crit Care.

[94] Zhou F, Peng Z, Murugan R, Kellum JA (2013). Blood purification and mortality in sepsis: a meta-analysis of randomized trials. Crit Care Med.

[95] Cruz DN, Antonelli M, Fumagalli R, Foltran F, Brienza N, Donati A, Malcangi V, Petrini F, Volta G, Bobbio Pallavicini FM, Rottoli F, Giunta F, Ronco C (2009). Early use of polymyxin B hemoperfusion in abdominal septic shock: the EUPHAS randomized controlled trial. JAMA.

[96] Damman K, van Deursen VM, Navis G, Voors AA, van Veldhuisen DJ, Hillege HL (2009). Increased central venous pressure is associated with impaired renal function and mortality in a broad spectrum of patients with cardiovascular disease. J Am Coll Cardiol.

[97] Mullens W, Abrahams Z, Francis GS, Sokos G, Taylor DO, Starling RC, Young JB, Tang WH (2009). Importance of venous congestion for worsening of renal function in advanced decompensated heart failure. J Am Coll Cardiol.

[98] Costanzo MR, Guglin ME, Saltzberg MT, Jessup ML, Bart BA, Teerlink JR, Jaski BE, Fang JC, Feller ED, Haas GJ, Anderson AS, Schollmeyer MP, Sobotka PA (2007). Ultrafiltration versus intravenous diuretics for patients hospitalized for acute decompensated heart failure. J Am Coll Cardiol.

[99] Bart BA, Goldsmith SR, Lee KL, Givertz MM, O’Connor CM, Bull DA, Redfield MM, Deswal A, Rouleau JL, LeWinter MM, Ofili EO, Stevenson LW, Semigran MJ, Felker GM, Chen HH, Hernandez AF, Anstrom KJ, McNulty SE, Velazquez EJ, Ibarra JC, Mascette AM, Braunwald E (2012). Ultrafiltration in decompensated heart failure with cardiorenal syndrome. N Engl J Med.

[100] Patarroyo M, Wehbe E, Hanna M, Taylor DO, Starling RC, Demirjian S, Tang WH (2012). Cardiorenal outcomes after slow continuous ultrafiltration therapy in refractory patients with advanced decompensated heart failure. J Am Coll Cardiol.

[101] Hemmer M, Viquerat CE, Suter PM, Vallotton MB (1980). Urinary antidiuretic hormone excretion during mechanical ventilation and weaning in man. Anesthesiology.

[102] Murugan R, Karajala-Subramanyam V, Lee M, Yende S, Kong L, Carter M, Angus DC, Kellum JA (2010). Acute kidney injury in non-severe pneumonia is associated with an increased immune response and lower survival. Kidney Int.

[103] Hoke TS, Douglas IS, Klein CL, He Z, Fang W, Thurman JM, Tao Y, Dursun B, Voelkel NF, Edelstein CL, Faubel S (2007). Acute renal failure after bilateral nephrectomy is associated with cytokine-mediated pulmonary injury. J Am Soc Nephrol.

[104] Dellinger RP, Levy MM, Rhodes A, Annane D, Gerlach H, Opal SM, Sevransky JE, Sprung CL, Douglas IS, Jaeschke R, Osborn TM, Nunnally ME, Townsend SR, Reinhart K, Kleinpell RM, Angus DC, Deutschman CS, Machado FR, Rubenfeld GD, Webb S, Beale RJ, Vincent JL, Moreno R (2013). Surviving Sepsis Campaign: international guidelines for management of severe sepsis and septic shock, 2012. Intensive Care Med.

[105] Forster C, Schriewer J, John S, Eckardt KU, Willam C (2013). Low-flow CO(2) removal integrated into a renal-replacement circuit can reduce acidosis and decrease vasopressor requirements. Crit Care.

[106] Burki NK, Mani RK, Herth FJ, Schmidt W, Teschler H, Bonin F, Becker H, Randerath WJ, Stieglitz S, Hagmeyer L, Priegnitz C, Pfeifer M, Blaas SH, Putensen C, Theuerkauf N, Quintel M, Moerer O (2013). A novel extracorporeal CO(2) removal system: results of a pilot study of hypercapnic respiratory failure in patients with COPD. Chest.

[107] Zacharia BE, Ducruet AF, Hickman ZL, Grobelny BT, Fernandez L, Schmidt JM, Narula R, Ko LN, Cohen ME, Mayer SA, Connolly ES (2009). Renal dysfunction as an independent predictor of outcome after aneurysmal subarachnoid hemorrhage: a single-center cohort study. Stroke.

[108] Corral L, Javierre CF, Ventura JL, Marcos P, Herrero JI, Manez R (2012). Impact of non-neurological complications in severe traumatic brain injury outcome. Crit Care.

[109] Liu M, Liang Y, Chigurupati S, Lathia JD, Pletnikov M, Sun Z, Crow M, Ross CA, Mattson MP, Rabb H (2008). Acute kidney injury leads to inflammation and functional changes in the brain. J Am Soc Nephrol.

[110] Ronco C, Bellomo R, Brendolan A, Pinna V, La Greca G (1999). Brain density changes during renal replacement in critically ill patients with acute renal failure. Continuous hemofiltration versus intermittent hemodialysis. J Nephrol.

[111] Davenport A, Will EJ, Davison AM (1990). Early changes in intracranial pressure during haemofiltration treatment in patients with grade 4 hepatic encephalopathy and acute oliguric renal failure. Nephrol Dial Transplant.

[112] Ko SB, Choi HA, Gilmore E, Schmidt JM, Claassen J, Lee K, Mayer SA, Badjatia N (2012). Pearls & Oysters: the effects of renal replacement therapy on cerebral autoregulation. Neurology.

[113] Benz-Worner J, Haberthur C, Kothbauer K (2011). Fluid and electrolyte management of acute traumatic brain injury using hemofiltration with regional citrate anticoagulation. J Neurosurg Anesthesiol.

[114] Kelleher JA, Chan TY, Chan PH, Gregory GA (1996). Protection of astrocytes by fructose 1,6-bisphosphate and citrate ameliorates neuronal injury under hypoxic conditions. Brain Res.

[115] Lindhoff-Last E, Betz C, Bauersachs R (2001). Use of a low-molecular-weight heparinoid (danaparoid sodium) for continuous renal replacement therapy in intensive care patients. Clin Appl Thromb Hemost.

[116] de Pont AC, Hofstra JJ, Pik DR, Meijers JC, Schultz MJ (2007). Pharmacokinetics and pharmacodynamics of danaparoid during continuous venovenous hemofiltration: a pilot study. Crit Care.

[117] Link A, Girndt M, Selejan S, Mathes A, Bohm M, Rensing H (2009). Argatroban for anticoagulation in continuous renal replacement therapy. Crit Care Med.

[118] Kiser TH, MacLaren R, Fish DN, Hassell KL, Teitelbaum I (2010). Bivalirudin versus unfractionated heparin for prevention of hemofilter occlusion during continuous renal replacement therapy. Pharmacotherapy.

[119] Kiser TH, Fish DN (2006). Evaluation of bivalirudin treatment for heparin-induced thrombocytopenia in critically ill patients with hepatic and/or renal dysfunction. Pharmacotherapy.

